# Comparison of Population-Based Association Study Methods Correcting for Population Stratification

**DOI:** 10.1371/journal.pone.0003392

**Published:** 2008-10-14

**Authors:** Feng Zhang, Yuping Wang, Hong-Wen Deng

**Affiliations:** 1 Institute of Molecular Genetics, School of Life Science and Technology, Xi'an Jiaotong University, Xi'an, Shaanxi, China; 2 Departments of Orthopedic Surgery and Basic Medical Science, School of Medicine, University of Missouri-Kansas City, Kansas City, Missouri, United States of America; 3 School of Computing and Engineering, University of Missouri-Kansas City, Kansas City, Missouri, United States of America; 4 Laboratory of Molecular and Statistical Genetics, College of Life Sciences, Hunan Normal University, Changsha, Hunan, China; University of Montreal, Canada

## Abstract

Population stratification can cause spurious associations in population–based association studies. Several statistical methods have been proposed to reduce the impact of population stratification on population–based association studies. We simulated a set of stratified populations based on the real haplotype data from the HapMap ENCODE project, and compared the relative power, type I error rates, accuracy and positive prediction value of four prevailing population–based association study methods: traditional case-control tests, structured association (SA), genomic control (GC) and principal components analysis (PCA) under various population stratification levels. Additionally, we evaluated the effects of sample sizes and frequencies of disease susceptible allele on the performance of the four analytical methods in the presence of population stratification. We found that the performance of PCA was very stable under various scenarios. Our comparison results suggest that SA and PCA have comparable performance, if sufficient ancestral informative markers are used in SA analysis. GC appeared to be strongly conservative in significantly stratified populations. It may be better to apply GC in the stratified populations with low stratification level. Our study intends to provide a practical guideline for researchers to select proper study methods and make appropriate inference of the results in population-based association studies.

## Introduction

Population-based association studies are powerful for gene mapping of complex diseases [1]–[3]. Population–based association studies detect non-random associations between alleles and disease status using unrelated individuals. Potential population stratification in unrelated sample may cause spurious positive or negative associations in population-based association studies [4]. Several statistical methods have been proposed to reduce the impact of population stratification on population-based association studies. These approaches include three major categories: structured association (SA) [5], [6], genomic control (GC) [7] and principal components analysis (PCA) [8]. SA is based on the presumption that population structure and individual ancestries can be estimated using a set of ancestral informative markers (AIMs) [5], [6]. We can then conduct association tests that condition on the inferred individual ancestries within each subpopulation. In the method of GC, it is assumed that original test statistics at all loci are inflated by population stratification in a similar way with a similar magnitude [7]. Therefore, the effect of population stratification can be assessed using a set of disease-unlinked marker loci, providing a correction factor that can then be applied to adjust for statistical bias at candidate loci. For PCA method, classical principal components analysis is first applied to genotype data to model ancestral differences between cases and controls, which are then used to correct allele frequency variations at candidate loci across ancestral populations [8]. Based on the corrected data, we can conduct association tests correcting for population stratification.

SA, GC and PCA all have been widely used in genetic studies [9]–[13]. An outstanding question, however, is the relative performance among these methods. Because of different hypotheses and algorithms, the performance and effectiveness under different situations of these methods may be different. By now, only limited comparisons have been conducted to evaluate and compare the relative performance of these methods to control for population stratification [14]–[16]. In these studies, not all of the methods aforementioned were studied. For example, the newest PCA method implemented in EIGENSOFT [8] was usually not studied. Furthermore, the previous studies compared the relative performance of these methods under only some limited parameter settings (e.g., range of sample sizes), which may limit the generality of their results.

In this study, we used the real haplotype data retrieved from the HapMap ENCODE project to simulate a set of stratified populations. We compared the relative performance among four prevailing population-based association study methods: traditional case-control test (TCCT), SA (implemented in STRUCTURE & STRAT), GC (implemented in EIGENSOFT) and PCA (implemented in EIGENSOFT) under various scenarios, considering population stratification levels, sample sizes, frequencies of disease susceptible allele and numbers of AIMs (for SA).

## Methods

### Data Simulation

We simulated a set of stratified populations based on the real haplotype data from the HapMap ENCODE project. The HapMap ENCODE project genotyped dense sets of SNPs across ten 500 kb regions in four populations. Phased haplotype data of Caucasians with northern and western European ancestry (CEPH) and Yoruba from Ibadan (YRI) of Africa were downloaded from HapMap ENCODE website (http://www.HapMap.org/downloads/phasing/2005-03_phaseI/ENCODE/). Within each ENCODE region, we selected the set of informative marker loci, which were genotyped in both CEPH and YRI, and were either polymorphic in at least one population or monomorphic, but had different alleles in the two populations. 12,867 informative marker loci were finally selected from the 10 HapMap ENCODE regions. Using the Kosambi map function [17], recombination fractions between adjacent informative marker loci were converted from the genetic map distances reported by the HapMap ENCODE project.

Based on the CEPH and YRI haplotype data and derived recombination fractions for informative marker loci, we first simulated the haplotype of 2000 CEPH and 2000 YRI as founder populations. CEPH and YRI founders were then separately and randomly mating for 20 generations to generate two discrete subpopulations. During this process, we assumed that all markers were under Hardy-Weinberg equilibrium and randomly recombined according to the derived recombination fractions. Population size was kept constant in each subpopulation. Finally, the simulated CEPH and YRI subpopulations were mixed together to generate a structured population.

Specific for this study, we randomly selected 240 of 12,867 informative marker loci evenly distributed across the 10 ENCODE regions with D'<0.3 in the simulated CEPH and YRI subpopulations for each simulation [18]. Allele frequencies at each of the 240 loci were recorded respectively in the simulated CEPH and YRI subpopulations, and in the final structured populations. The causal locus used to simulate individual disease phenotype was randomly selected from the 240 loci with preset allele frequency in the simulated structured populations and different allele frequencies in the simulated CEPH and YRI subpopulations (0.20<frequency difference<0.30), to ensure the existence of population stratification at the causal locus in the simulated structured populations.

Single causal gene additive model was implemented to generate individual disease status in simulated CEPH and YRI subpopulations, respectively [19]. We assumed that a bi-allelic locus was associated with disease status. The relationship among population prevalence (K), genotype relative risk (GRR) (r), allele frequency (p) and penetrance of genotypes at the causal locus (f_i_) in the simulated structured populations can be expressed as
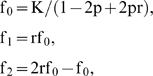
where f_i_ denotes the penetrance of the genotypes at the causal locus with i copy (copies) of disease susceptible allele (i = 0, 1 or 2). The GRR was assigned 2.0 or 1.0 to simulate the causal locus with or without genetic effect on individual disease status. The population prevalence (K) was assigned 0.01 in all models. Equivalent numbers of cases and controls were randomly drawn from the simulated structured populations for each parameter setting. The above simulation procedure was repeated until sufficient cases and controls were obtained.

### Parameter design

To compare the relative performance of the four association study methods in the presence of population stratification, we simulated a set of populations under various stratification parameters to model no, low, moderate and high degrees of population stratification. Stratification levels were controlled by sampling cases and controls from the simulated CEPH and YRI subpopulations with different proportions [15]. In addition, we assessed the effects of sample sizes and frequencies of disease susceptible allele on the performance of the four analytical methods in the stratified populations with high stratification level. The parameter designs are presented in Table 1.

**Table 1:** Parameter configurations in the simulation studies.

Stratification levels^a^	Sample sizes^b^	FDSA^c^	Numbers of AIMs
0.30−0.30,	400	0.10±0.02	**40**
0.35−0.25	800	**0.20±0.02**	80
0.40−0.20	**1200**	0.30±0.02	120
**0.50−0.10**	2000	0.40±0.02	200

Note: ^a^ denote the proportions of YRI individuals in cases-controls, respectively.

b denote the numbers of total samples comprising of equivalent cases and controls.

c denote the frequencies of disease susceptible allele.

d The basic parameter configuration is highlighted in bold. Each possible parameter setting can be obtained by replacing one entry of the basic parameter configuration with a different entry of corresponding parameter.

Due to the extensive computational cost required by SA (1000 simulations using 120 AIMs need at least 30 days of computing time for each parameter setting at our computer cluster), we initially selected 40 of 240 informative marker loci as AIMs to assess the performance of SA. To investigate the effect of numbers of AIMs on the performance of SA, we further conducted SA analysis using various numbers of AIMs (Table 1).

### Data analysis

1000 simulations were conducted for each parameter setting. In each simulation, all 240 loci were analyzed by chi-square tests (TCCT) and EIGENSOFT, which conducted both GC and PCA tests. For GC, correction factor λ was first estimated by EIGENSOFT using 239 loci (excluding the causal locus). The estimated correction factor λ was then used to adjust the statistics at the causal locus. For SA tests implemented in STRUCTURE & STRAT, 40 of 240 loci were first randomly selected from 9 ENCODE regions (excluding the region containing the causal locus) with minor allele frequencies>0.01 in the simulated structured populations. We calculated the average allele frequency difference at the 40 loci between the simulated CEPH and YRI subpopulations. In 1000 simulations, the average allele frequency difference was 0.117 (standard deviation = 0.116). The 40 loci were analyzed by STRUCTURE to infer individual ancestries as AIMs. The remaining 200 loci were then analyzed by STRAT to conduct association tests incorporating the inferred population structure by STRUCTURE. All the above software was running under default parameters recommended by the program developers.

In each simulation, positive result was defined as *P* value< = 0.05 obtained at the causal locus. Power and type I error rates were calculated respectively as the proportions of positive results obtained at the causal locus with (GRR = 2.0) and without (GRR = 1.0) phenotypic effect in 1000 simulations. Additionally, because positive results can be caused by population stratification except for true disease-locus association [4], it is not reasonable to directly compare the power of these analytical methods, which have different type I error rates. To precisely evaluate the relative performance of the four analytical methods, we further calculated accuracy and positive prediction value (PPV) for each method. Positive and negative results obtained at the causal locus with phenotypic effect (GRR = 2.0) were regarded as true positive (TP) and false negative (FN), respectively. Similarly, positive and negative results obtained at the causal locus without phenotypic effect (GRR = 1.0) were regarded as false positive (FP) and true negative (TN), respectively. For each parameter setting, in 2000 simulations (1000 simulations for power and 1000 simulations for type I error rates), accuracy and PPV were derived as
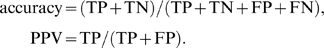



## Results

**Figure 1:**
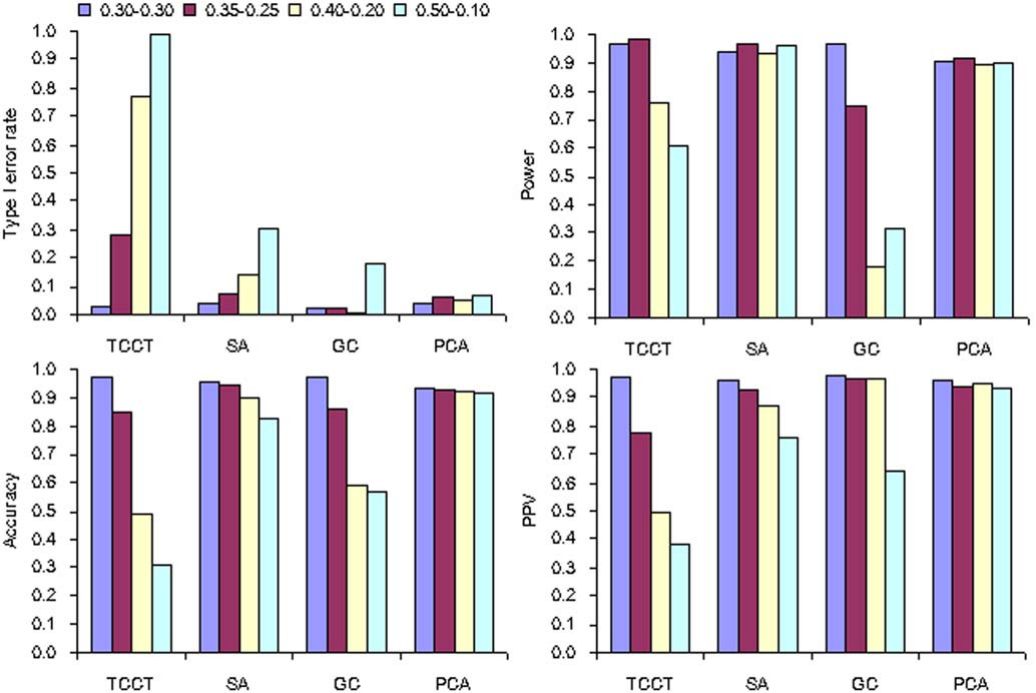
Performance of the four analytical methods in stratified populations with stratification levels varying from 0.3−0.3 to 0.5−0.1 (sample size = 1200, frequency of disease susceptible allele = 0.20±0.02 and number of AIMs = 40).

The comparison results of the four association study methods under different scenarios are summarized in figures 1∼[Fig pone-0003392-g002] [Fig pone-0003392-g003] 4. It is obvious that the performance of all analytical methods is affected by various parameters investigated here. The effects of each parameter on the performance of the four analytical methods are detailed in the following:

**Figure 2 pone-0003392-g002:**
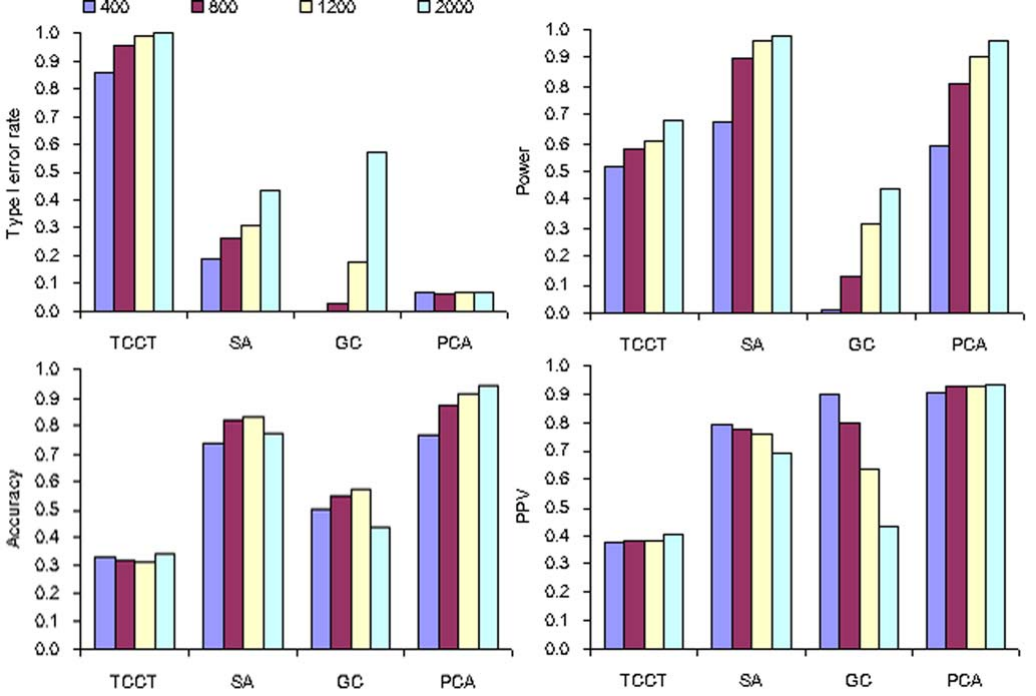
Performance of the four analytical methods in stratified populations with sample sizes varying from 400 to 2000 (stratification level = 0.5−0.1, frequency of disease susceptible allele = 0.20±0.02 and number of AIMs = 40).

**Figure 3 pone-0003392-g003:**
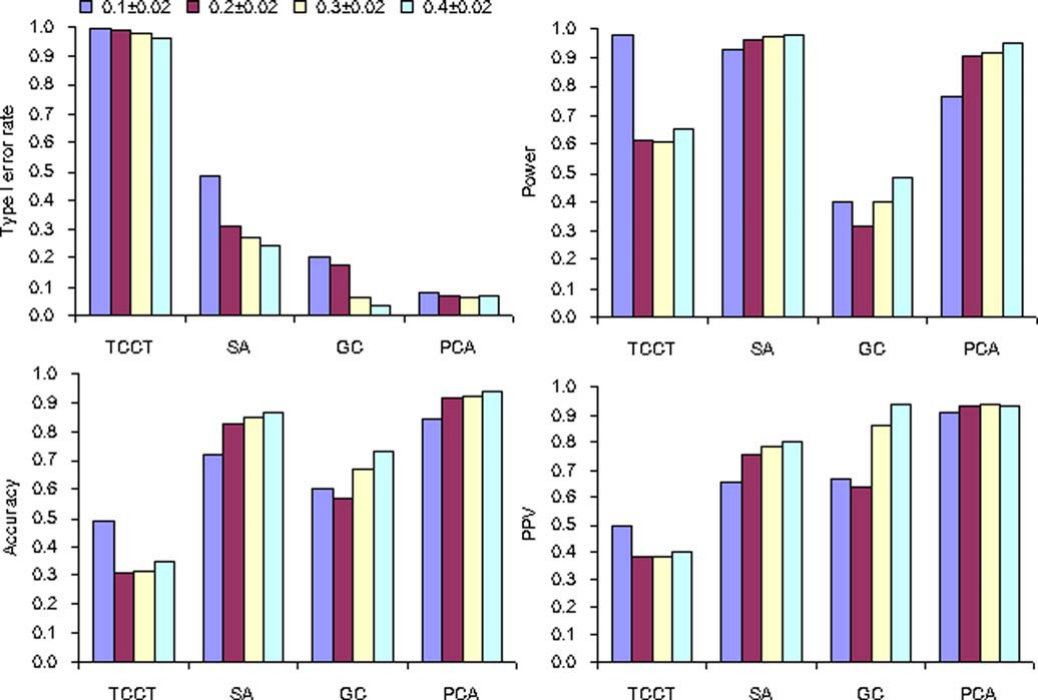
Performance of the four analytical methods in stratified populations with frequencies of disease susceptible allele varying from 0.10±0.02 to 0.40±0.02 (stratification level = 0.5−0.1, sample size = 1200 and number of AIMs = 40).

**Figure 4:**
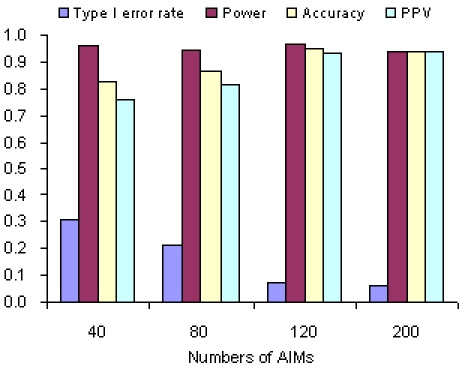
Performance of SA with numbers of AIMs varying from 40 to 200 (stratification level = 0.5−0.1, sample size = 1200 and frequency of disease susceptible allele = 0.20±0.02).

### Stratification levels

Table 2 summarizes the average correction factor λ estimated by GC approach under various stratification levels. λ was larger than 2.8 in the presence of population stratification, and was greatly increased with increasing stratification levels. Figure 1 presents the performance of the four analytical methods under various stratification levels. As previously reported, the performance of TCCT was significantly affected by population stratification. The type I error rates of TCCT were much higher than that of SA, GC and PCA at the same stratification level. For accuracy and PPV, TCCT showed significantly decreasing trends with increasing stratification levels. SA and PCA outperformed GC in almost all performance indexes except for type I error rates and PPV in the stratified populations with low and moderate stratification levels. With increasing stratification levels, SA showed significant increase in type I error rates as well as decreases in accuracy and PPV, which were not observed in PCA.

**Table 2:** Average corrector factor λ estimated by GC in populations with various stratification levels.

Stratification levels^a^	λ	
	Power^b^	Type I error rates^c^
0.30−0.30	1.10	1.04
0.35−0.25	2.98	2.85
0.40−0.20	7.56	7.51
0.50−0.10	11.93	11.91

Note: ^a^ denote the proportions of YRI individuals in cases-controls, respectively.

b were calculated from power comparison results.

c were calculated form type I error rate comparison results.

### Sample sizes

With the increase of sample sizes from 400 to 2000, both power and type I error rates tended to increase in all analytical methods except for PCA. The type I error rates of PCA were not significantly inflated by larger sample sizes (Figure 2). Compared with SA, PCA yielded higher accuracy and PPV under the same sample sizes.

### Frequencies of disease susceptible allele

Figure 3 provides an overview of comparison results with respect to frequencies of disease susceptible allele. With the increase of frequencies of disease susceptible allele from 0.01±0.02 to 0.04±0.02, we observed a consistent decrease of type I error rates in all analytical methods except for PCA, which attained much lower type I error rates than other analytical methods. Compared with SA, PCA also performed better in accuracy and PPV under the same frequencies of disease susceptible allele.

### Numbers of AIMs

As shown by figure 4, the type I error rates of SA significantly decreased with increasing numbers of AIMs, and became close to 0.05 when 120 or more AIMs were used. In contrast, the power of SA was not significantly affected by numbers of AIMs.

## Discussion

In the simulation study for stratification levels, we observed significant adverse influence of population stratification on TCCT, which has been reported by previous studies [20]. Among the three analytical methods against population stratification, SA and PCA generally outperformed GC. It is impressive that the performance of PCA was very stable under various stratification levels, indicating its good ability in controlling for population stratification. Additionally, the computational cost required by PCA is much smaller than that of SA [8]. We observed a high type I error rate for SA in the stratified populations with high stratification level, which may be explained by the small set of AIMs that we used. Increasing the numbers of AIMs significantly decreased the type I error rates of SA. Considering the similar performance between SA and PCA when 120 or more AIMs were used, we believe that SA and PCA have comparable performance if sufficient AIMs are applied in SA analysis. However, due to the extensive computational cost required by SA, we suggest that PCA should be a better choice to conduct population-based association studies correcting for population stratification, especially in the studies that need analyze large amounts of genetic markers.

In our study, GC performed worse than SA and PCA in most situations. In the stratified populations with low stratification level, GC attained the lowest type I error rate and moderately lower power than SA and PCA, which is consistent with previous observation [15]. In the stratified populations with moderate and high stratification levels, the average correction factor λ estimated by GC became very large (>7.5). After the adjustment of λ, the power of GC was much lower than that of SA and PCA. GC appeared to be strongly conservative in significantly stratified populations, which has been reported by previous studies [14], [16]. The loss of power for GC may be attributed to the hypothesis in GC that the original statistics at both candidate loci and null loci were affected by population stratification in a similar way with a similar magnitude [7], which may result in an overestimate of λ for candidate loci, especially in significantly stratified populations [14]. Based on the results of our and other previous studies [14], [16], we suggest that the performance of GC correcting for population stratification is affected by population stratification levels. It may be better to apply GC in slightly stratified populations.

Another interesting finding is the masking effects of population stratification on true disease-gene associations [4]. With increasing stratification levels, we simultaneously observed decreasing power and increasing type I error rates. It has be suggested that population stratification can cause not only false positive associations, but also false negative associations [4]. Our result confirms that population stratification can result in a loss of power through masking true disease-gene associations in addition to causing false positive associations. It should be careful to explain negative associations yielded from population-based association studies in the presence of population stratification.

Sample sizes and frequencies of disease susceptible allele are the two other major factors affecting the accuracy of gene mapping. For sample sizes, we found that the impact of population stratification on population–based association studies tended to increase with increasing sample sizes. Our result confirms the observation from previous study that larger sample sizes may not only lead to higher power, but also higher type I error rates in the presence of population stratification [21]. The increase of type I error rates may partially be explained by increasing genetic heterogeneity in larger sample. Additionally, the performance of the four analytical methods was significantly affected by frequencies of disease susceptible allele. Within the frequencies range of disease susceptible allele that we investigated, lower type I error rates and higher accuracy and PPV were generally observed with larger frequencies of disease susceptible allele.

Four aspects from our study deserve further emphasized. First, to the best of our knowledge, all of existing performance comparison studies directly calculated the positive results obtained at candidate loci with and without phenotypic effect as power and type I error rates, respectively [14]–[16]. The potential problem in above design is that the positive results can not only be attributed to true disease-loci associations, but also to the false positive results caused by population stratification [4]. This makes it difficult to precisely evaluate and compare the relative performance of different analytical methods, which have different type I error rates. To overcome this limitation, in our study, we further calculated accuracy and PPV for each method, which can provide more accurate information about the performance of the four association study methods. For example, as shown by figure 1, the power of TCCT significantly decreased with increasing stratification levels in the presence of population stratification, which can partly be explained by the increase of false negative results caused by population stratification. Therefore, directly using power to evaluate the performance of different analytical methods may lead to inaccurate conclusion in the presence of population stratification. Second, we used the real CEPH and YRI haplotype data from the HapMap ENCODE project to simulate structured populations. The simulated data sets are closer to realistic scenario compared with existing similar simulation studies [14]–[16], which ensures the robustness and practical applicability of our results. Additionally, compared with the genome-wide data available in the HapMap, the ENCODE regions were identified with approximately tenfold higher density of SNPs in extensive resequencing data, which enables the ENCODE data to provide richer and more accurate information about genetic variation across different populations [22]. Third, admixture model is another common population structure model in addition to the discrete model we used here [23]. Due to the difficulty of controlling population stratification levels under the admixture model, we evaluated the performance of the four analytical methods in stratified populations without admixture. The discrete model is extensively used to simulate stratified populations [8], [24]. The study results under the discrete model can be generally extended to more complex conditions, such as stratified populations with admixture. Fourth, because of extensive computational cost, we randomly selected 40 of 240 informative marker loci as AIMs to compare SA with other methods. In practice, we can use more AIMs, which will improve the performance of SA. Selecting a set of AIMs with maximizing ancestral information is an alternative for improving the performance of SA. For instance, AIMs can be selected to maximize absolute allele frequency differences among different ancestral populations [25]. However, these methods require prior knowledge about individuals' membership or ancestries to known populations, which are usually not available or not certain in practice.

In summary, we compared the relative performance among four prevailing association study methods under various scenarios. Our efforts can provide a practical guideline for researchers to select proper study methods and make appropriate interpretation of the results in population-based association studies.
